# Challenges, Difficulties, and Delayed Diagnosis of Multiple Myeloma

**DOI:** 10.3390/diagnostics15131708

**Published:** 2025-07-04

**Authors:** Tugba Zorlu, Merve Apaydin Kayer, Nazik Okumus, Turgay Ulaş, Mehmet Sinan Dal, Fevzi Altuntas

**Affiliations:** Department of Hematology & Apheresis Unit, Ankara Oncology Training and Research Hospital, University of Health Sciences, Ankara 06200, Türkiye; tbazrlu@gmail.com (T.Z.); apaydinmerve@yahoo.com (M.A.K.); nzk.okumus@gmail.com (N.O.); dr.sinandal@gmail.com (M.S.D.); faltuntas@hotmail.com (F.A.)

**Keywords:** artificial intelligence, diagnostic delay, multiple myeloma, monoclonal gammopathy of undetermined significance, misdiagnosis

## Abstract

**Background:** Multiple myeloma (MM) is a heterogeneous plasma cell malignancy with non-specific symptoms and disease heterogeneity at clinical and biological levels. This non-specific set of symptoms, including bone pain, anemia, renal failure, hypercalcemia, and neuropathy, can mislead diagnosis as chronic or benign conditions, resulting in a delay in diagnosis. Timely identification is paramount to prevent organ damage and reduce morbidity. **Methods:** In this review, we present an overview of recent literature concerning the factors leading to the delayed diagnosis of MM and the impact of delayed diagnosis. This includes factors relevant to physicians and systems, diagnostic processes, primary healthcare services, and laboratory and imaging data access and interpretation. Other emerging technologies to diagnose NCIs include AI-based decision support systems and biomarker-focused strategies. **Findings:** Delayed diagnosis can lead to presentation at advanced disease stages associated with life-threatening complications and shorter progression-free survival. Patients are often seen by many physicians before they are referred to hematology. Understanding of clinical red flags for MM in primary care is inadequate. Our findings indicate that limited access to diagnostic tests, inconsistent follow-up of MGUS/SMM patients, and a lack of interdepartmental coordination delay the diagnostic process. **Conclusions:** Multimodal tools for early diagnosis of MM. Educational campaigns to raise awareness of the disease, algorithms dedicated to routine care and novel technologies, including AI and big data analytics, and new biomarkers may serve this purpose, as well as genomic approaches to the premalignant MGUS stage.

## 1. Introduction

**Figure 1:**
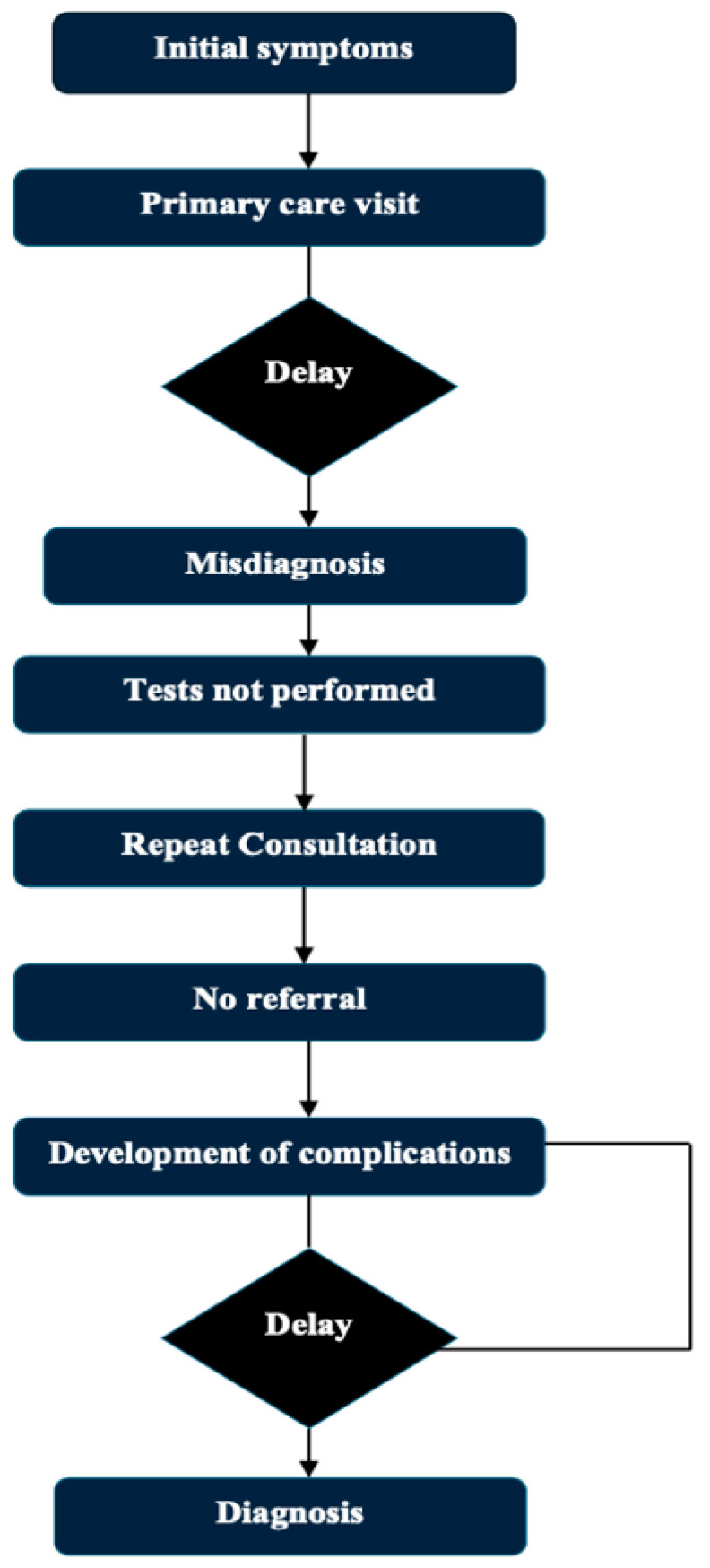
Typical diagnostic journey in MM and delay points.

Multiple myeloma (MM) is a hematological cancer derived from plasma cells, often defined by the presence of a monoclonal immunoglobulin. It accounts for 1% of cancers and about 10% of hematological malignancies. And findings during the course of the disease may include anemia, leukopenia, thrombocytopenia, renal failure, severe pain, bone fractures, and hypercalcemia [1,2,3,4]. However, almost all multiple myelomas arise from an asymptomatic premalignant condition called monoclonal gammopathy of undetermined significance (MGUS). This is diagnosed after more than 10 years of the disorder in 50% [5,6,7]. Effective multi-drug regimens are available for most forms of MM, resulting in long-term remission. However, because of the disease’s biology, relapse remains frequent, and MM remains incurable due to the emergence of treatment resistance [8,9,10]. MM has variable clinical findings, non-specific symptoms, as seen in diabetes, arthritis, or chronic renal failure, which can all lead to a late diagnosis [11], especially in elderly patients, where there are many differential diagnoses mimicking MM. MGUS affects ~3% of individuals over age 50, while SMM is found in 0.5–1%. In comparison, hypertension and diabetes affect ~40% and ~25% of the same population, respectively [11]. Patients often develop these symptoms and present to primary healthcare providers or other specialists, giving rise to delays in referrals to hematologists. Delayed diagnosis of the disease can negatively impact patients’ quality of life and prognosis [12]. The routine path to diagnosis of multiple myeloma and possible delays is presented in Figure 1. Existing reviews and articles on this topic are sparse. The objective of this review is to present and evaluate the multifactorial factors which create and perpetuate delays in the diagnoses of multiple myeloma, highlight barriers that exist at the patient, physician, and system perspective level, present potential strategies that may facilitate earlier diagnoses and earlier interventions, and provide supplemental data to fill gaps in the existing literature to foster academic progress in this area.

To the best of our knowledge, there is currently no systematic integrative review that combines diagnostic delays to multiple myeloma with system gaps in healthcare, AI-guided approaches, or biomarker-related strategies. This gap is addressed in the present manuscript, which offers an interdisciplinary synthesis covering clinical, diagnostic, and technological fields.

## 2. Literature Search Strategy

A scoping literature review was conducted to evaluate studies addressing diagnostic dilemmas and delays in multiple myeloma (MM), early detection strategies, and the use of artificial intelligence (AI) and biomarker-based tools. The search was performed in PubMed, Embase, and the Cochrane Library covering the period from January 2010 to March 2024.

The following keywords and Boolean combinations were used: “multiple myeloma” AND (“diagnostic delay” OR “delayed diagnosis”) AND (“artificial intelligence” OR “biomarkers” OR “early detection” OR “MGUS” OR “smoldering myeloma”).

Only peer-reviewed original articles, meta-analyses, clinical trials, and systematic reviews published in English were included. Studies focusing on adult populations, reporting on diagnostic pathways, delays, outcomes, and systemic barriers to early diagnosis were prioritized.

Titles and abstracts were screened independently by two reviewers, and full-text evaluation was performed for eligible articles. After removing duplicates, a total of 85 studies were included in the final synthesis.

## 3. Clinical Spectrum of Multiple Myeloma

The symptoms in multiple myeloma are usually non-specific. Its defining symptoms are the CRAB symptoms (hypercalcemia, renal insufficiency, anemia, and bone findings) [1,2,3,4]. Among 1027 multiple myeloma patients included in the Czech post-marketing observational study, bone pain was reported at diagnosis by 58% of the patients, and fatigue by 32% of the patients. A total of 73% of the patients had anemia, 48% had elevated creatinine levels, 13% had hypercalcemia, and 79% had bone abnormalities (primarily but not limited to lytic lesions, fractures, and osteoporosis). Other features typically noted are neuropathy, developing infections (recurrent), loss of weight (involuntary), and easy bruising/bleeding [13].

Because most symptoms are non-specific, they can resemble many other conditions. Diabetes shares some symptoms with multiple myeloma, including excessive thirst and urination, fatigue, frequent infections, and neuropathy [14]. Likewise, renal sufficiency due to multiple myeloma is difficult to separate from renal insufficiency from diabetes or chronic kidney disease in the absence of directed testing [15]. Bone pain or low back pain is frequently shrugged off as arthritis or osteoporosis and not taken seriously. This results in delayed diagnosis, which consequently leads to increased morbidity and mortality [16]. The most frequent presenting clinical symptoms for multiple myeloma, along with potential misunderstandings and diagnostic delays, are outlined in Table 1.

It has been shown that patients consult at least three specialists before arriving at a hematologist, which can cause a lapse of 3 to 6 months in the diagnostic workup [17]. Delayed diagnosis is linked with a higher rate of myeloma-related complications and a significant decrease in disease-free survival, but it does not affect the overall survival rate [18]. Worse outcomes have also been associated with presenting with advanced complications (e.g., severe infections, spinal cord compression, fractures, and renal failure) [19].

## 4. Reasons for Delays in Diagnosis

### 4.1. Patient-Related Delay

The median age of presentation for MM is 70 years [20], and most patients are elderly. Symptoms like chronic pain and fatigue are often misattributed to natural ageing or ignored completely by patients, resulting in a delay in diagnosis [21]. Symptoms such as anemia, bone pain, and fatigue are often thought of as a normal part of daily life, leading to delayed consultation with a physician [22]. This delays the process of diagnosis and adds to the probability of the disease being detected at an advanced stage with the associated complications. Diagnostic challenges, such as consideration of atypical presentations including extramedullary disease in MM, also result in delays in the diagnosis [23].

### 4.2. Physician-Related Delays

The non-specific nature of multiple myeloma symptoms, together with the long asymptomatic phase that can precede the disease’s onset, leads to diagnostic delay, both due to patient factors and also because physicians frequently will not suspect the condition [24]. Commonest symptoms such as fatigue, back pain, anemia, and recurrent infections are often ascribed to age or to non-malignant conditions [25]. In particular, in primary care settings, general practitioners are often more likely to exclude multiple myeloma and pursue the most commonly encountered diagnoses, such as depression, osteoarthritis, or viral illness, resulting in delays in making the correct diagnosis that can be many months in duration [26].

The above-mentioned determinants reinforcing such delay, as revealed by a United Kingdom study addressing the pre-diagnostic phase, also included physicians attributing symptoms to either “muscle pain” or “age-related issues”, neglecting lab abnormalities, restricting physical examination or negligence of series of preclinical steps (the process before a diagnosis) [27]. Moreover, the limited knowledge base of many physicians leads to other myeloma-specific findings (such as elevated erythrocyte sedimentation rate (ESR), normochromic normocytic anemia, hypercalcemia) being attributed to benign conditions [28]. Delays in diagnosis not only result in the disease being detected at an advanced stage but also lead to decreased trust in the healthcare system and unnecessary hospital visits [29]. Therefore, rasing awareness among primary care physicans about multiple myeloma and ensuring timely referrals to secondary care when necessary are curial steps toward early diagnosis [30].

Extramedullary paraskeletal involvement is noted in about 13% of patients: during diagnosis in 7% and an additional 6% during treatment. This is because these involvements typically manifest as localized complaints, leading to the consideration of hematological malignancy being missed [31].

Back pain and anemia are not unusual in a primary care population but are not, in and of themselves, reasons to consider multiple myeloma. Accordingly, we modified the diagnostic algorithm to favor the combination of red flag features. These consist of normocytic anemia without obvious cause, elevated ESR, hypercalcemia, bone pain refractory to analgesia, or renal failure. The objective is to assist general practitioners in recognizing patients who need further hematological workup, and at the same time, avoid overreferrals or excessive testing.
Symptom/FindingNotesPersistent back or bone painEspecially if not responding to analgesicsNormocytic normochromic anemiaHb < 10 g/dL, no clear causeElevated ESR or CRPESR > 60 mm/h may raise suspicionHypercalcemiaCa > 11 mg/dLUnexplained fatigue and weight lossConstitutional symptomsRenal impairmenteGFR < 60 mL/min with no other causeMonoclonal protein in SPEP/UPEPIf performed incidentally or due to above symptomsIf ≥ 2 criteria present → consider serum protein electrophoresis (SPEP), serum free light chains, and referral to hematology. Note: MM is rare. Avoid overtesting in young patients with isolated mechanical back pain or iron-deficiency anemia due to menstrual bleeding or GI losses.

### 4.3. System-Related Delays

In the laboratory, tests necessary for the diagnosis of multiple myeloma include a complete blood count (CBC), erythrocyte sedimentation rate (ESR), biochemistry, serum protein electrophoresis, and free light chain levels [1,2,3,4]. Although bone marrow biopsy is the key to a diagnosis, sophisticated imaging techniques, such as whole-body low-dose computed tomography (CT), positron emission tomography–computed tomography (PET-CT), and whole-body magnetic resonance imaging (MRI), are commonly needed to confirm a diagnosis. But the accessibility, interpretability, and coordination of these tests often cause delays [32].

Additionally, multiple myeloma is a genetically and biologically heterogeneous disease [10]. Aggressive molecular methods (Fluorescence In Situ Hybridization analysis, for instance) are paramount in properly identifying patients with high-risk subtypes but do not capture the whole palette of the disease at a molecular level [33]. Recently, new sequencing technologies (whole-genome sequencing, whole-exome sequencing, and panels) have been established for more accurate risk stratification to elucidate mutations behind drug resistance. The clinical integration of such novel analyses is still limited, and delays in diagnosis persist as an important clinical failure [34].

The disease’s clinical features and the potential treatment complications need multidisciplinary assessment between hematologists, laboratory assistants, genetic specialists, nephrologists, radiologists, neurologists, and sometimes neurosurgeons [35]. Nevertheless, a substantial amount of time can be lost during the diagnostic process if the communication and coordination between several specialties are not well established [36]. Table 2 lists some of the patient, physician, and system factors that are relevant to the diagnosis and treatment of multiple myeloma that may lead to delay. Table 3 shows the analysis of factors leading to diagnostic delay, considering physician-related and system-related factors.

#### 4.3.1. Tests and Limitations in the Diagnostic Process

Given that the diagnosis of MM is often made late in the course of the disease process, it is imperative that primary care physicians begin the diagnostic algorithm when they observe any of the aforementioned symptoms (e.g., fatigue, weight loss) or abnormalities on routine laboratory testing (e.g., anemia, elevated creatinine, hypercalcemia, elevated ESR, or elevated total protein levels [37]. In this setting, relatively simple and directed tests like serum protein electrophoresis (SPEP), serum free light chain (sFLC) testing, and serum immunofixation electrophoresis (IFE) should be the first tests requested [38,39]. At this stage of imaging, a low-dose CT whole-body scan, if available, is to be preferred due to its sensitivity in the detection of osteolytic lesions. The alternative methods that can be used, not covered by CT, are advanced methods like PET-CT or MRI, and the request for these tests is usually made by a hematologist or an oncologist [40] (Table 4). Hence, early referral of suspected cases with necessary lab tests from primary care to hematology aids in quicker diagnosis and avoids complications [40]. Widespread implementation of diagnostic algorithms in primary care, better awareness among general practitioners, and establishing rapid referral chains are required to prevent delays. The diagnosis is then made according to the International Myeloma Working Group Diagnostic Criteria for Multiple Myeloma and Certain Related Plasma Cell Disorders [35] once a hematologist has completed the workup.

#### 4.3.2. Laboratory and Imaging Methods Used in the Diagnosis of Multiple Myeloma

Laboratory and imaging diagnostic techniques for multiple myeloma report varying sensitivity and specificity for the identification of unique subtypes because of the biological heterogeneity of the disease [41]. Although serum protein electrophoresis and immunofixation tests are the most common techniques to identify monoclonal immunoglobulins, they are known to have low sensitivity, especially in light chain-only (LCO) or non-secretory myeloma [38,42]. In these subgroups, the serum free light chain (sFLC) test is of clear diagnostic utility, indirectly providing evidence of monoclonality due to alterations in the kappa and lambda ratio [43]. However, approximately 20% of LCO patients have non-elevated FLC that cannot be reliably identified in urine, thus limiting the utility both in diagnosis and treatment response assessment, while above 100 mg/L offers a significant gain in reliability [44].

However, many advanced diagnostic methods are unavailable in all healthcare facilities. Technologies such as mass spectrometry and isoelectric focusing that are more sensitive are still not routinely used and are only available to a handful of centers due to the expense, infrastructure, and personnel required. For imaging, techniques such as low-dose whole-body CT, PET-CT, and whole-body MRI are essential for the early detection of osteolytic lesions but often rely on hematologist/oncologist referrals [45]. Moreover, advanced radiological experience is needed for the interpretation of these tests, and risks, like interpretational variability or false negatives, can add to the challenge in diagnosis [46]. Table 5 provides a summary of the main diagnostic techniques for MM and their sensitivity, specificity, and limitations.

In addition, traditional methodologies like Fluorescence In Situ Hybridization analysis can detect high-risk cytogenetic abnormalities but cannot provide a complete picture of molecular heterogeneity in multiple myeloma at the molecular level [47]. Next-generation sequencing technologies (whole-genome or whole-exome sequencing) overlap the ability to probe more deeply into the genetic infrastructure of the disease, but these techniques have not yet gained broad translation into clinical practice due to barriers of cost, interpretation of data, and a lack of infrastructure; thus, they are limited in the application to the vast majority of MM patients. Collectively, these factors represent major technical and systematic obstacles towards accurate, rapid, and subtype-specific diagnosis of multiple myeloma [34]. New diagnostics in MM in the last several years have also seen the emergence of new biomarkers and technology for improvement in earlier and more accurate diagnosis of MM, as shown in Table 6. Although widely used, many of these diagnostic tests have deficiencies with regard to sensitivity, specificity, or application, as listed in Table 7.

## 5. Consequences of Early Diagnosis

These challenges result in patients being diagnosed later, receiving treatment more slowly, and experiencing worse clinical outcomes [48]. Late identification raises the risk of potentially fatal issues in patients [48]. This results in increased morbidity and mortality, leading to more aggressive initial treatments, which drive up costs [49]. Those who present with renal failure at diagnosis have limited therapeutic options, and those who present with vertebral fractures have limited mobility, chronic pain, and reduced quality of life [50]. Moreover, the higher demand for treatment associated with late-stage disease manifests as an increased number of patients requiring hospitalization and intensive care, and it has an impact on the healthcare system, which, in turn, has economic implications [51]. Delays in diagnosis also lead to more long-term problems, including lower treatment compliance, lower quality of life, loss of workforce productivity, and psychosocial effects [52]. Hence, early diagnosis of the disease not only has clinical but also economic and social significance in multiple myeloma [53]. Table 8 provides the clinical and prognostic implications of delayed diagnosis in multiple myeloma.

## 6. Strategies to Prevent Diagnostic Delays

Diagnostic interval in multiple myeloma is one of the longest in all types of cancer that adversely affect both patient outcomes and a burden on the healthcare system [36]. Such delays often occur in primary care, since general practitioners (GPs) rarely observe this rare disease clinically [54]. Moreover, an average GP will see multiple myeloma only once every 8–10 years, making it very unlikely to be suspected early on [26]. Hence, the enhancement of a GP’s familiarity with myeloma symptomatology and diagnostic pathways, as well as the implementation of diagnostic safety nets for persistent and unexplained symptoms, remains a key strategic priority [55].

Early diagnosis may easily be established through the recognition of minor abnormalities in routine blood tests, along with the implementation of reflex myeloma screening [56]. Clinical risk algorithms created for this approach seek to identify high-risk patients based on their electronic health records, utilizing parameters including symptoms, hemoglobin, creatinine, and inflammatory markers [57] There are technical challenges that still exist with it being integrated into decision support systems, including, alignment with clinical workflows, and figuring out triggering thresholds. Active surveillance of precursor conditions such as MGUS and smoldering multiple myeloma (SMM) is also potentially valuable [58]. A population-based screening study conducted in Iceland provides one valuable model, during which all MGUS patients were studied, applying different follow-up strategies [59].

**Figure 2:**
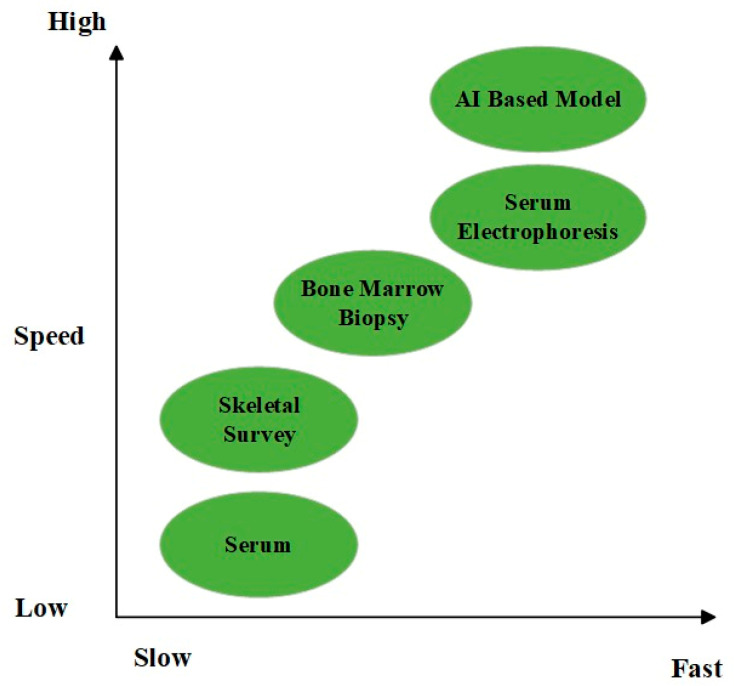
Comparison of diagnostic tools in terms of speed and sensitivity.

In addition, working with laboratories on early warning systems and proactive collaboration with hematologists can help facilitate diagnoses, particularly in less obvious cases. To facilitate timely referral decisions, critical test results must be interpreted according to pre-defined algorithms and communicated to the clinician with the correct reflex tests [60]. In addition to their reliance on technological integration, cost-effectiveness, sustainability of their impact on the healthcare system, and diagnostic accuracy must be used to assess early diagnostic strategies. This approach can enhance the early detection of myeloma at a much earlier stage with minimal complications and improve basic clinical endpoints, such as survival [61]. A graphic comparison of two chosen diagnostic tools according to their speed and sensitivity is presented in Figure 2.

Early diagnosis of MM may lead to the prevention of end-organ damage, but it is unclear whether this also affects overall survival (OS). Some investigations (e.g., Mateos et al., 2018) indicate that there is no OS benefit, likely emphasizing the relevance of disease biology and cytogenetic profile as more impactful to outcomes than diagnosis timing in general [62].

The AQUILA trial was a randomized phase III trial investigating the clinical benefit of early treatment in high-risk SMM and was reported in NEJM in 2024. The trial included more than 200 patients who were considered high-risk based on the percentage of bone marrow plasma cells, ratio of serum free light chains, and levels of M-protein. The patients were assigned to up-front, lenalidomide-based therapy or observation. Results The results indicated that the early therapy group had a statistically significant PFS benefit compared with the observation group. But there was no significant difference in OS at the time of data cutoff. Crucially, the organ-protective potential for early intervention seemed restricted. Although there were fewer complications of anemia in the treatment group, there were no significant differences between the two groups in the incidence of bone disease or renal failure. These results indicate that early treatment may postpone disease progression, but that its effect on longer-term outcomes, such as OS and irreversible organ damage, is unknown, which further argues for a personalized treatment strategy in high-risk SMM patients [63].

Although published cumulative prospective data correlating such delays with survival from multiple myeloma is scant, the studies that have been published show an overwhelming association between late diagnosis and the endpoint of irreversible end-organ damage. For example, Gonsalves et al. (2015) [64] demonstrated that 21% of the newly diagnosed MM patients had renal failure (defined as serum creatinine ≥ 2 mg/dL), of which 58% fell into ISS stage III at diagnosis. By comparison, just 29% of individuals who did not have renal failure received their diagnosis at this late stage. While diagnostic delay duration was not explicitly addressed by the study, these results suggest that longer symptomatic duration before diagnosis can result in silent disease progression and ensuing renal dysfunction and more aggressive presenting disease. As renal impairment in MM typically progresses gradually, this supports the clinical benefit for early identification and referral in patients with no obvious cause for anemia, energy, or an elevated creatinine [64]. The association between diagnostic delay and complication onset in multiple myeloma (MM) is complicated. In a previous large retrospective study of 5483 patients using Medicare-SEER linked data, the authors reported a median interval of diagnosis of 99 days after clinical presentation, most often manifested as anemia or back pain. Interestingly, bone pain was reported in 47.5% of patients up to two years before diagnosis, with a median delay of 220 days (interquartile range (IQR) of 80 to 476) from the first symptom of bone pain to the MM diagnosis. Contrary to the belief that long-term delay may contribute to skeletal or renal complications, multivariate analysis revealed that diagnostic delay in itself was not an independent risk factor for complications (OR 0.9, 95% CI: 0.8–1.1). Complicated rather than non-complicated lesions were more closely related to indicators of disease severity and co-morbidities. However, the results also underscore that nearly half of MM patients presented with early musculoskeletal symptoms; however, imaging or appropriate diagnostic workup was not performed in a significant number, with only 60% undergoing imaging in the setting of bone symptoms. This highlights the need for clinical awareness in patients with unknown bone pain, anemia, and renal involvement, particularly in elderly patients [24,29]. As summarized in Table 9, several studies have investigated the association between diagnostic delay and clinical complications in MM.

While the discovery of precursor conditions such as MGUS and SMM theoretically can identify who will ultimately progress to active disease, identification and early intervention are currently controversial. Bianchi et al. (Blood 2010) noted that the finding of MGUS in screening or other settings (e.g., incidental discovery during workup for some nonsymptomatic matter) might result in overdiagnosis and unnecessary counseling, testing, or procedures for the condition [65].

In addition, the current iStopMM (Iceland Screens, Treats, or Prevents Multiple Myeloma) trial is designed to determine the value of population-wide screening, although final results are pending, and to date, no data exist to support routine screening practices [62].

## 7. Future Perspectives and Research Areas

Sufficient studies on enhanced diagnostic and prognostic markers are ongoing due to the rising incidence of multiple myeloma (MM) cases and the burdens and delays in diagnosis [66]. MiRNAs, angiogenic factors, Extracellular Matrix proteins, telomeres, and telomerase activities are newer biomarkers with promising capabilities in the diagnostics and prognostics of MM [67]. Ongoing studies on methodologies such as blood biopsy, which could permit earlier disease detection than the still gold-standard invasive bone marrow biopsy but still allow more frequent and less painful measurements through the variants in circulating tumor cells (CTCs), miRNAs, and cell-free circulating DNA (cfDNA) in peripheral circulation [68].

As with other sectors, there is a growing use of artificial intelligence in medicine. As the number of cases of multiple myeloma increases, so does the volume of data that has been gathered [69]. Such data can be processed using machine learning and deep learning models to expand the knowledge and better understand myeloma mechanisms to better manage MM patients [70,71].

AI-based diagnostic support systems have held great promise in the early detection of multiple myeloma (MM) and its precursor states. Some recent works have used machine learning (ML) models on routine laboratory results to increase diagnostic performance. For instance, Fan et al. (2022) constructed a random forest classifier model combining CBC and metabolic panel information, with an AUC of 0.968 and an accuracy of 92.6% for differentiation of MGUS and active MM [72]. Similarly, Allegra et al. (2022) reviewed several deep learning-based approaches and reported that convolutional neural networks (CNNs) applied to bone marrow aspirate images and clinical datasets achieved diagnostic accuracies exceeding 90% [70]. These results highlight that AI tools could be integrated into electronic medical records to serve as early alert systems, especially for patients with non-specific symptoms like fatigue or anemia. Concurrently, biology-based approaches, including the sFLC ratio, B2M, and bone-marrow-derived B-cell maturation antigen, are gaining more traction as potential diagnostic adjuncts. Instead, emerging markers such as BCMA have the potential to be prioritized in both diagnosis and therapeutic targets, like sFLC and beta-2 microglobulin, which are part of the current diagnostic criteria. But, in spite of encouraging retrospective results, there is a paucity of prospective validation and real-world adoption for these technologies, particularly for resource-limited health systems [70,72]. These findings suggest that AI-powered tools can be effectively integrated into electronic medical records as early alert systems, particularly for patients presenting with non-specific symptoms such as fatigue or anemia. In parallel, biology-driven approaches—such as the serum free light chain (sFLC) ratio, beta-2 microglobulin (B2M), and bone marrow–derived B-cell maturation antigen (BCMA)—are also gaining prominence as valuable diagnostic adjuncts. Emerging markers like BCMA not only offer diagnostic utility but also serve as potential therapeutic targets, similar to sFLC and B2M, which are already part of current diagnostic criteria. However, despite promising retrospective data, there remains a significant lack of prospective validation and limited real-world application of these technologies, particularly in resource-limited healthcare systems.

Despite the potential shown by AI models and biomarkers for multiple myeloma, there are several obstacles to their real-world use. First, many AI models that show high diagnostic accuracy have been trained and tested on retrospective data, often in artificial academic settings. Even if an algorithm is already ready for use in primary care or community oncology, this often necessitates standardized data entry, tight integration with electronic health records, and trained clinicians. These resources may be scarce beyond the walls of a large academic medical center. In addition, many healthcare systems lack developed frameworks for the regulatory approval of AI-assisted diagnostics, leading to a legal vacuum around clinical responsibility. The same is true for biomarkers like serum free light chains and BCMA, which are both diagnostically and prognostically useful. However, cost, laboratory platform variation, and the lack of established clinical cutoffs for screening in the absence of symptoms limit implementation. These limitations are magnified in low- and middle-income countries where resource constraints, laboratory infrastructure, and reimbursement policies serve as even more onerous barriers. Thus, while AI and biomarker approaches may be promising, their application in the real world will require prospective testing, clinician education, economic evaluation, and policy reform [73]. Table 10 summarizes the performance characteristics of selected AI models that have been evaluated for the early detection of multiple myeloma.

MGUS (monoclonal gammopathy of undetermined significance) is a prolonged premalignant phase prior to the development of multiple myeloma [74]. This phase allows for the study of cancer evolution, for understanding plasma cell neoplasm malignant evolution mechanisms, for identifying MGUS patients with a high risk of progression early, and for eventually designing new therapeutic targets considering previous achievements. This may also delay or extend the process of malignant transformation [75,76].

The application of rapid advances in genomic techniques to study how premalignant cells evolve should also allow for the identification and subsequent targeting of driver events during clonal evolution in MM and in cancer more broadly [76,77].

Recent advancements have led to the development of AI-driven models aimed at predicting MM risk from routine clinical data. For instance, an XGBoost algorithm trained on 200 electronic health record (EHR) parameters achieved an AUC of 0.84 for 5-year MM risk prediction in a large Clalit dataset (*n* = 4256 cases vs. 20:1 controls) [78]. A simplified logistic regression model from the same study, using only 20 laboratory variables, also showed promising performance (AUC 0.72) [78]. In addition, natural language processing (NLP) models have been applied to unstructured clinical notes, enabling earlier detection of MGUS/MM cases by several months [79].

Regarding biomarkers, elevated serum levels of DKK1—linked to skeletal disease—have been proposed as diagnostic markers in MM; capillary electrophoresis assays identified optimal cutoff values (approximately 10 ng/mL) for clinical use [80]. Moreover, DKK1 and sclerostin—both Wnt pathway inhibitors—correlated with the extent of bone disease and decreased significantly post-treatment [81]. Although these biomarkers are not yet part of standard diagnostic criteria, they hold potential both for disease monitoring and for guiding therapeutic decisions.

These additions provide concrete evidence and real-world metrics, strengthening the narrative and addressing reviewer concerns about the lack of specific AI and biomarker examples.

## 8. Conclusions

Multiple myeloma (MM) is the second most common hematological malignancy, yet its diagnosis is frequently delayed due to non-specific symptoms, lack of awareness, and multifactorial barriers involving patients, physicians, and healthcare systems. The majority of MM cases are diagnosed at an advanced stage, increasing morbidity and reducing patients’ quality of life. Although microbiological, serological, and imaging techniques have advanced, their widespread implementation remains limited by standardization issues and resource constraints.

This review highlights multiple avenues for early diagnosis, including raising awareness through educational programs, development of algorithm-based approaches for primary care, integration of artificial intelligence (AI) tools, and the use of biomarker-based risk assessment—especially during the MGUS premalignant phase. Removing obstacles to early diagnosis may effectively reduce morbidity and late-stage complications. However, current evidence does not clearly support a direct correlation between early diagnosis and reduced early mortality, as emphasized by recent studies and ongoing trials (e.g., iStopMM).

Moreover, the early detection of low-risk MGUS, particularly in younger individuals, may lead to overdiagnosis, increased patient anxiety, and unnecessary follow-up or overtreatment. These psychosocial and healthcare burdens should be considered when developing population-based screening strategies. Although guidelines exist for the follow-up of MGUS and SMM, it is not yet proven that such monitoring prevents progression to symptomatic MM or organ damage.

The average interval between initial symptoms and a confirmed MM diagnosis ranges from 6 to 11 months in the literature, with delays exceeding 3 months being associated with higher rates of complications such as anemia or bone disease.

This review contributes to the literature by providing a structured synthesis of diagnostic delays in MM, supported by recent data, and integrating evolving diagnostic approaches including AI and biomarkers. By exposing the interplay between clinical uncertainty, systemic barriers, and delayed recognition, our study offers both practical suggestions and research perspectives that can support earlier and more effective diagnosis strategies in real-world settings.

## Figures and Tables

**Table 1:** Common symptoms of MM and diagnostic pitfalls.

Symptom	MM Association	Misdiagnosis Risk	Delay Potential
Back pain	High	Osteoporosis, arthritis	High
Fatigue/anemia	High	Aging, depression	High
Polyuria/polydipsia	Moderate	Diabetes mellitus	Moderate
Renal insufficiency	High	Hypertensive nephropathy	High

**Table 2:** Factors contributing to diagnostic delays in MM.

Level	Examples	References
Patient-level	Attributing symptoms to aging, neglecting chronic pain	[20,21,22,23]
Physician-level	Misdiagnosing non-CRAB symptoms, labeling as depression	[24,25,26,27,28,29,30,31]
System-level	Delayed imaging, late referral to hematology	[32,33,34,35,36]

**Table 3:** Physician vs. system-related delay factors.

Delay Factor	Physician-Related	System-Related
Symptom interpretation	Mislabeling as benign/age-related	Lack of diagnostic pathway algorithms
Test ordering	Not requesting SPEP, sFLC early	Delay in test availability (MRI, PET-CT)
Follow-up	Inadequate follow-up on anemia/ESR	Poor interdepartmental communication
Referral	Delayed referral to hematology	Lack of fast-track hematology referral

**Table 4:** IMWG diagnostic criteria for MM and related disorders.

MGUS (non-IgM ^a^)
All 3 criteria must be met: Serum monoclonal protein (non-IgM type) < 3 g/dL Clonal bone marrow plasma cells < 10% ^b^ Absence of end-organ damage such as hypercalcemia, renal insufficiency, anemia, and bone lesions (CRAB) or amyloidosis that can be attributed to the plasma cell proliferative disorder
SMM
Both criteria must be met: Serum monoclonal protein (IgG or IgA) of ≥3 g/dL, or urinary monoclonal protein ≥ 500 mg/24 h, or clonal bone marrow plasma cells 10–60% Absence of MDEs or amyloidosis
MM
Both criteria must be met: Clonal bone marrow plasma cells ≥ 10% or biopsy-proven bony or extramedullary plasmacytoma ≥1 of the following MDEs: Evidence of end-organ damage that can be attributed to the underlying plasma cell proliferative disorder, specifically ▪ Hypercalcemia: serum calcium > 1 mg/dL higher than the ULN or >11 mg/dL ▪ Renal insufficiency: CrCl < 40 mL/min or serum creatinine > 2 mg/dL ▪ Anemia: Hgb of >2 g/dL below the LLN or <10 g/dL ▪ Bone lesions: ≥1 osteolytic lesion(s) on skeletal radiography, CT, or PET-CT ⸰ Clonal bone marrow plasma cell percentage ≥ 60% ⸰ Involved:uninvolved sFLC ratio ≥100 (involved FLC level must be ≥100 mg/L) ⸰ >1 focal lesions on MRI (at least 5 mm in size)
Solitary plasmacytoma
All 4 criteria must be met: Biopsy-proven solitary lesion of bone or soft tissue with evidence of clonal plasma cells Normal bone marrow with no evidence of clonal plasma cells Normal skeletal survey and MRI (or CT) of spine and pelvis (except for the primary solitary lesion) Absence of end-organ damage such as hypercalcemia, renal insufficiency, anemia, or bone lesions (CRAB) that can be attributed to a lympho-plasma cell proliferative disorder

CrCl = creatinine clearance; CT = computed tomography; FLC = free light chain; Hgb = hemoglobin; LLN = lower limit of normal; MDEs = myeloma-defining events; MGUS = monoclonal gammopathy of undetermined significance; MM = multiple myeloma; MRI = magnetic resonance imaging; PET-CT = positron emission tomography-computed tomography; sFLC = serum free light chain; SMM = smoldering multiple myeloma; ULN = upper limit of normal. SI conversion factors: To convert serum calcium to mmol/L, multiply values by 0.25; to convert serum creatinine to μmol/L, multiply values by 88.4; to convert Hgb to g/L, multiply values by 10. ^a^ For diagnostic criteria associated with other types of MGUS (ie, IgM MGUS and light-chain MGUS), see Rajkumar et al., 2014. [35] ^b^ Bone marrow biopsy can be deferred in patients with low-risk MGUS (IgG-type, M protein < 15 g/L, normal FLC ratio) who lack clinical features concerning for MM [41]. The underlined letters represent the acronym CRAB (hyperCalcemia, Renal insufficiency, Anemia, Bone lesions).

**Table 5:** Diagnostic tools and their characteristics.

Method	Sensitivity	Specificity	Use Case	Limitation
SPEP	Medium	High	Detects M protein	Ineffective in non-secretory MM
Sflc	High	Medium	Light chain disease	Complex interpretation
Bone Marrow Biopsy	High	High	Gold standard	Invasive
Whole-body MRI	High	High	Detects marrow lesions early	Limited access
PET-CT	High	High	Extramedullary/osseous involvement	Cost and availability
Mass Spectrometry	Very High	Very High	Low-level M-protein detection	Limited to specialized centers

**Table 6:** Emerging biomarkers and technologies.

Marker/Technology	Diagnostic Value	Current Limitation
miRNA	Early marker of transformation	Not yet routine
circulating DNA	Minimally invasive detection	Expensive, technical complexity
circulating tumor cells	Monitoring progression	Low abundance, technical challenges
AI-based algorithms	Risk stratification and early alerting	Not yet fully integrated into systems
Mass Spectrometry	Ultra-sensitive M-protein detection	Requires expertise and infrastructure

**Table 7:** Diagnostic tests and limitations in MM.

Method	Advantages	Limitations
Serum protein electrophoresis	Inexpensive, widely available	Insufficient in non-secretory MM
sFLC assay	Valuable in light-chain only MM	Interpretation complexity
Bone marrow biopsy	Gold standard	Invasive, difficult to repeat
Whole-body MRI	Effective for lytic lesions	Limited accessibility

**Table 8:** Consequences of delayed diagnosis.

Consequence	Description	Example
Late-stage diagnosis	Diagnosis occurs after irreversible organ damage	45% with bone disease at diagnosis
Increased complications	Higher rates of renal failure, fractures, spinal cord compression	21% renal failure at diagnosis
Need for aggressive treatment	More toxic regimens required at presentation	Induction with triplet/quad regimens more common in ISS III
Reduced treatment options	Limited options in renal failure or frail patients	Lenalidomide contraindicated in CrCl < 30 mL/min
Decreased quality of life	Pain, fatigue, and mobility limitations	Chronic bone pain in >60% at diagnosis
Higher healthcare costs	Increased burden on hospital and outpatient care	US MM patients: ~USD 120 k/year
Increased hospitalizations	More ICU admissions and procedure burden	1.5× ICU utilization in delayed diagnosis
Psychosocial impact	Depression, anxiety, loss of function/productivity	20–30% with clinically significant anxiety

**Table 9:** Selected findings on diagnostic delay and associated complications in MM.

Clinical Indicator	Median Delay (Days)	Rate of Finding (%)	Reference
First bone pain → MM diagnosis	220	47.5% with bone symptoms	Ramasamy et al. (2021) [24]
Anemia prior to diagnosis	73	65.6% evaluated for Hb	Ramasamy et al. (2021) [24]
Renal dysfunction prior to diagnosis	58	74.1% evaluated for creatinine	Ramasamy et al. (2021) [24]
Renal failure at diagnosis	—	21% of patients	Gonsalves et al. (2015) [64]

**Table 10:** Performance characteristics of selected AI models for early MM detection.

Study (Year)	Model Type	Diagnostic Target	Input Data Type	Accuracy (%)	AUC
Fan et al. (2022) [72]	Random Forest	MGUS vs. MM	CBC + metabolic panel	92.6%	0.968
Allegra et al. (2022) [70]	Convolutional Neural Net	Early MM detection	Retrospective EHR	>90%	-
Clinical assessment [1]	Physician judgment	Routine clinical evaluation (history, labs)	Clinical observation	Limited diagnostic accuracy	-

## References

[1] Rajkumar S.V. (2020). Multiple myeloma: 2020 update on diagnosis, risk-stratification and management. Am. J. Hematol..

[2] Malard F., Neri P., Bahlis N.J., Terpos E., Moukalled N., Hungria V.T.M., Manier S., Mohty M. (2024). Multiple myeloma. Nat. Rev. Dis. Primers.

[3] Vrtis M.C. (2024). Multiple Myeloma. Home Healthc. Now.

[4] Rajkumar S.V. (2024). Multiple myeloma: 2024 update on diagnosis, risk-stratification, and management. Am. J. Hematol..

[5] Landgren O., Kyle R.A., Pfeiffer R.M., Katzmann J.A., Caporaso N.E., Hayes R.B., Dispenzieri A., Kumar S., Clark R.J., Baris D. (2009). Monoclonal gammopathy of undetermined significance (MGUS) consistently precedes multiple myeloma: A prospective study. Blood.

[6] Kyle R.A., Therneau T.M., Rajkumar S.V., Larson D.R., Plevak M.F., Offord J.R., Dispenzieri A., Katzmann J.A., Melton L.J.I. (2006). Prevalence of monoclonal gammopathy of undetermined significance. N. Engl. J. Med..

[7] Weiss B.M., Abadie J., Verma P., Howard R.S., Kuehl W.M. (2009). A monoclonal gammopathy precedes multiple myeloma in most patients. Blood.

[8] Kumar S., Htut M., Sharman J.P. (2020). Efficacy of four-drug regimens in multiple myeloma: A systematic review. Blood.

[9] Moreau P., Kumar S.K., San Miguel J., Davies F., Zamagni E., Bahlis N., Ludwig H., Mikhael J., Terpos E., Schjesvold F. (2021). Treatment of relapsed and refractory multiple myeloma: Recommendations from the International Myeloma Working Group. Lancet Oncol..

[10] Manier S., Salem K.Z., Park J., Landau D.A., Getz G., Ghobrial I.M. (2017). Genomic complexity of multiple myeloma and its clinical implications. Nat. Rev. Clin. Oncol..

[11] Smith D., Yong K. (2013). Multiple myeloma. BMJ.

[12] Koshiaris C., Oke J., Abel L., Nicholson B.D., Ramasamy K., Bruel A.V.D. (2018). Quantifying intervals to diagnosis in myeloma: A systematic review and meta-analysis. BMJ Open.

[13] Kyle R.A., Remstein E.D., Therneau T.M., Dispenzieri A., Kurtin P.J., Hodnefield J.M., Larson D.R., Plevak M.F., Jelinek D.F., Fonseca R. (2007). Clinical course and prognosis of smoldering (asymptomatic) multiple myeloma. N. Engl. J. Med..

[14] Ali M.A., Ahmed Y.A., Ibrahim A. (2013). Clinical challenges: Myeloma and concomitant type 2 diabetes. South Asian J. Cancer.

[15] Dimopoulos M.A., Sonneveld P., Leung N., Merlini G., Ludwig H., Kastritis E., Goldschmidt H., Joshua D., Orlowski R.Z., Powles R. (2016). International Myeloma Working Group Recommendations for the Diagnosis and Management of Myeloma-Related Renal Impairment. J. Clin. Oncol..

[16] Dimopoulos M.A., Kastritis E. (2008). The role of novel drugs in multiple myeloma. Ann Oncol..

[17] Mohty M., Cavo M., Fink L., Gonzalez-McQuire S., Leleu H., Mateos M., Raab M.S., Schoen P., Yong K. (2019). Understanding mortality in multiple myeloma: Findings of a European retrospective chart review. Eur. J. Haematol..

[18] Howell D., Smith A., Appleton S., Bagguley T., Macleod U., Cook G., Patmore R., Roman E. (2017). Multiple myeloma: Routes to diagnosis, clinical characteristics and survival—Findings from a UK population-based study. Br. J. Haematol..

[19] Kazandjian D., Mailankody S., Korde N., Landgren O. (2014). Smoldering multiple myeloma: Pathophysiologic insights, novel diagnostics, clinical risk models, and treatment strategies. Clin. Adv. Hematol. Oncol..

[20] Howlader N., Noone A.M., Krapcho M., Miller D., Brest A., Yu M., Ruhl J., Tatalovich Z., Mariotto A., Lewis D.R. (2021). SEER Cancer Statistics Review, 1975–2018.

[21] Urban V.S., Cegledi A., Mikala G. (2023). Multiple myeloma, a quintessential malignant disease of aging: A geroscience perspective on pathogenesis and treatment. Geroscience.

[22] Kariyawasan C.C., Hughes D.A., Jayatillake M.M., Mehta A.B. (2007). Multiple myeloma: Causes and consequences of delay in diagnosis. QJM.

[23] Bladé J., Beksac M., Caers J., Jurczyszyn A., von Lilienfeld-Toal M., Moreau P., Rasche L., Rosiñol L., Usmani S.Z., Zamagni E. (2022). Extramedullary disease in multiple myeloma: A systematic literature review. Blood Cancer J..

[24] Seesaghur A., Petruski-Ivleva N., Banks V.L., Wang J.R., Abbasi A., Neasham D., Ramasamy K. (2021). Clinical features and diagnosis of multiple myeloma: A population-based cohort study in primary care. BMJ Open.

[25] Loberg M., Aas T., Wist E., Kiserud C.E. (2018). Symptoms and diagnostic delay in multiple myeloma. Tidsskr. Nor. Legeforening.

[26] Koshiaris C., Van den Bruel A., Oke J.L., Nicholson B.D., Shephard E., Braddick M., Hamilton W. (2018). Early detection of multiple myeloma in primary care using blood tests: A case-control study in primary care. Br. J. Gen. Pract..

[27] LeBlanc M.R., LeBlanc T.W., Leak Bryant A., Pollak K.I., Bailey D., Smith S. (2021). A Qualitative Study of the Experiences of Living With Multiple Myeloma. Oncol. Nurs. Forum.

[28] Negrete-Rodríguez P., Gallardo-Pérez M.M., Lira-Lara O., Melgar-de-la-Paz M., Hamilton-Avilés L.E., Ocaña-Ramm G., Robles-Nasta M., Sánchez-Bonilla D., Olivares-Gazca J.C., Mateos M.-V. (2024). Prevalence and Consequences of a Delayed Diagnosis in Multiple Myeloma: A Single Institution Experience. Clin. Lymphoma Myeloma Leuk..

[29] Friese C.R., Abel G.A., Magazu L.S., Neville B.A., Richardson L.C., Earle C.C. (2009). Diagnostic delay and complications for older adults with multiple myeloma. Leuk. Lymphoma.

[30] Hossain I., Hampson P., Nowell C., Khan S., Sen R., Sundararaman S., Adiyodi J., Basu S. (2021). An in Depth Analysis of Factors Contributing to Diagnostic Delay in Myeloma: A Retrospective UK Study of Patients Journey from Primary Care to Specialist Secondary Care. Blood.

[31] Pour L., Sevcikova S., Greslikova H., Kupska R., Majkova P., Zahradova L. (2014). Soft-tissue extramedullary multiple myeloma prognosis is significantly worse in comparison to bone-related extramedullary relapse. Haematologica.

[32] Dimopoulos M.A., Moreau P., Terpos E., Mateos M.V., Zweegman S., Cook G., Delforge M., Hájek R., Schjesvold F., Cavo M. (2021). Multiple myeloma: EHA-ESMO Clinical Practice Guidelines for diagnosis, treatment and follow-up. Ann Oncol..

[33] Ahmann G.J., Chng W.J., Henderson K.J., Price-Troska T.L., DeGoey R.W., Timm M.M., Dispenzieri A., Greipp P.R., Sable-Hunt A., Bergsagel L. (2008). Effect of tissue shipping on plasma cell isolation, viability, and RNA integrity in the context of a centralized good laboratory practice-certified tissue banking facility. Cancer Epidemiol. Biomark. Prev..

[34] Lohr J.G., Stojanov P., Carter S.L., Cruz-Gordillo P., Lawrence M.S., Auclair D., Sougnez C., Knoechel B., Gould J., Saksena G. (2014). Widespread genetic heterogeneity in multiple myeloma: Implications for targeted therapy. Cancer Cell.

[35] Rajkumar S.V., Dimopoulos M.A., Palumbo A., Blade J., Merlini G., Mateos M.V., Kumar S., Hillengass J., Kastritis E., Richardson P. (2014). Multiple myeloma: International Myeloma Working Group guidelines for diagnosis, treatment, and response. Lancet Oncol..

[36] Lyratzopoulos G., Abel G.A., McPhail S., Neal R.D., Rubin G.P. (2013). Measures of promptness of cancer diagnosis in primary care: Secondary analysis of national audit data on patients with 18 common and rarer cancers. Br. J. Cancer.

[37] Kumar S.K., Dispenzieri A., Lacy M.Q., Gertz M.A., Buadi F.K., Pandey S., Kapoor P., Dingli D., Hayman S.R., Leung N. (2014). Continued improvement in survival in multiple myeloma: Changes in early mortality and outcomes in older patients. Leukemia.

[38] Dispenzieri A., Kyle R.A., Katzmann J.A., Therneau T.M., Larson D., Benson J., Clark R.J., Melton L.J., Gertz M.A., Kumar S.K. (2008). Immunoglobulin free light chain ratio is an independent risk factor for progression of smoldering (asymptomatic) multiple myeloma. Blood.

[39] Dispenzieri A., Katzmann J.A., Kyle R.A., Larson D.R., Melton L.J., Colby C.L., Therneau T.M., Clark R., Kumar S.K., Bradwell A. (2010). Prevalence and risk of progression of light-chain monoclonal gammopathy of undetermined significance: A retrospective population-based cohort study. Lancet.

[40] Hillengass J., Usmani S., Rajkumar S.V., Durie B.G.M., Mateos M.-V., Lonial S., Joao C., Anderson K.C., García-Sanz R., Riva E. (2019). International myeloma working group consensus recommendations on imaging in monoclonal plasma cell disorders. Lancet Oncol..

[41] Boyd K.D., Ross F.M., Walker B.A., Wardell C.P., Tapper W.J., Chiecchio L., Dagrada G., Konn Z.J., Gregory W.M., NCRI Haematology Oncology Studies Group (2011). Mapping of chromosome 1p deletions in myeloma identifies FAM46C at 1p12 and CDKN2C at 1p32.3 as being genes in regions associated with adverse survival. Clin. Cancer Res..

[42] Murray D., Kumar S.K., Kyle R.A., Dispenzieri A., Dasari S., Larson D.R., Vachon C., Cerhan J.R., Rajkumar S.V. (2019). Detection and prevalence of monoclonal gammopathy of undetermined significance: A study utilizing mass spectrometry-based monoclonal immunoglobulin rapid accurate mass measurement. Blood Cancer J..

[43] Katzmann J.A., Clark R.J., Abraham R.S., Bryant S., Lymp J.F., Bradwell A.R., Kyle R.A. (2002). Serum reference intervals and diagnostic ranges for free kappa and free lambda immunoglobulin light chains: Relative sensitivity for detection of monoclonal light chains. Clin. Chem..

[44] Bradwell A.R., Carr-Smith H.D., Mead G.P., Harvey T.C., Drayson M.T. (2003). Serum test for assessment of patients with Bence Jones myeloma. Lancet.

[45] Rajkumar S.V., Landgren O., Mateos M.V. (2015). Smoldering multiple myeloma. Blood.

[46] Zamagni E., Nanni C., Patriarca F., Englaro E., Castellucci P., Geatti O., Tosi P., Tacchetti P., Cangini D., Perrone G. (2007). A prospective comparison of 18F-fluorodeoxyglucose positron emission tomography-computed tomography, magnetic resonance imaging and whole-body planar radiographs in the assessment of bone disease in newly diagnosed multiple myeloma. Haematologica.

[47] Avet-Loiseau H., Attal M., Moreau P., Charbonnel C., Garban F., Hulin C., Leyvraz S., Michallet M., Yakoub-Agha I., Garderet L. (2007). Genetic abnormalities and survival in multiple myeloma: The experience of the Intergroupe Francophone du Myélome. Blood.

[48] Quinn S.C., Kelly L., Lewis T. (2023). The Additional Disease Burden Accompanying a Delayed Diagnosis of Multiple Myeloma: Utilising a Large Real-World Evidence Base. Blood.

[49] Costa L.J., I Gonsalves W., Kumar S. (2014). Early Mortality in Multiple Myeloma: Risk Factors and Impact on Population Outcomes. Blood.

[50] Dimopoulos M.A., Kastritis E., Rosinol L., Bladé J., Ludwig H. (2008). Pathogenesis and treatment of renal failure in multiple myeloma. Leukemia.

[51] Kansal A., Latour J.M., See K.C., Rai S., Cecconi M., Britto C., Morris A.C., Savio R.D., Nadkarni V.M., Rao B.K. (2023). Interventions to promote cost-effectiveness in adult intensive care units: Consensus statement and considerations for best practice from a multidisciplinary and multinational eDelphi study. Crit. Care.

[52] Religioni U., Barrios-Rodríguez R., Requena P., Borowska M., Ostrowski J. (2025). Enhancing Therapy Adherence: Impact on Clinical Outcomes, Healthcare Costs, and Patient Quality of Life. Medicina.

[53] Yong C., Korol E., Khan Z., Lin H.M., Thompson J., Valovicova V., Luptakova K., Seal B. (2016). A real-world evidence systematic literature review of health-related quality of life, costs and economic evaluations in newly-diagnosed multiple myeloma patients. Blood.

[54] Green T., Atkin K., Macleod U. (2015). Cancer detection in primary care: Insights from general practitioners. Br. J. Cancer.

[55] Lyratzopoulos G., Vedsted P., Singh H. (2015). Understanding missed opportunities for more timely diagnosis of cancer in symptomatic patients after presentation. Br. J. Cancer.

[56] Smith L., Carmichael J., Cook G., Shinkins B., Neal R.D. (2022). Diagnosing myeloma in general practice: How might earlier diagnosis be achieved?. Br. J. Gen. Pract..

[57] Shephard E.A., Neal R.D., Rose P., Walter F.M., Litt E.J., Hamilton W.T. (2015). Quantifying the risk of multiple myeloma from symptoms reported in primary care patients: A large case-control study using electronic records. Br. J. Gen Pract..

[58] Rajkumar S.V. (2016). Updated Diagnostic Criteria and Staging System for Multiple Myeloma. Am. Soc. Clin. Oncol. Educ. Book.

[59] Rögnvaldsson S., Love T.J., Thorsteinsdottir S., Reed E.R., Óskarsson J.Þ., Pétursdóttir Í., Sigurðardóttir G.Á., Viðarsson B., Önundarson P.T., Agnarsson B.A. (2021). Iceland screens, treats, or prevents multiple myeloma (iStopMM): A population-based screening study for monoclonal gammopathy of undetermined significance and randomized controlled trial of follow-up strategies. Blood Cancer J..

[60] Cowan A.J., Allen C., Barac A., Basaleem H., Bensenor I., Curado M.P., Foreman K., Gupta R., Harvey J., Hosgood H.D. (2018). Global Burden of Multiple Myeloma: A Systematic Analysis for the Global Burden of Disease Study 2016. JAMA Oncol..

[61] Kazandjian D. (2016). Multiple myeloma epidemiology and survival: A unique malignancy. Semin. Oncol..

[62] Mateos M.V., Hernández M.T., Giraldo P., de la Rubia J., de Arriba F., Lopez Corral L., Rosinol L., Paiva B., Palomera L., Bargay J. (2013). Lenalidomide plus dexamethasone for high-risk smoldering multiple myeloma. N. Engl. J. Med..

[63] Dimopoulos M.A., Voorhees P.M., Schjesvold F., Cohen Y.C., Hungria V., Sandhu I., Lindsay J., Baker R.I., Suzuki K., Kosugi H. (2025). Daratumumab or Active Monitoring for High-Risk Smoldering Multiple Myeloma. N. Engl. J. Med..

[64] Gonsalves W.I., Leung N., Rajkumar S.V., Dispenzieri A., Lacy M.Q., Hayman S.R., Buadi F.K., Dingli D., Kapoor P., Go R.S. (2015). Improvement in renal function and its impact on survival in patients with newly diagnosed multiple myeloma. Blood Cancer J..

[65] Bianchi G., Kyle R.A., Larson D.R., Witzig T.E., Kumar S., Dispenzieri A., Morice W.G., Rajkumar S.V. (2013). High levels of peripheral blood circulating plasma cells as a specific risk factor for progression of smoldering multiple myeloma. Leukemia.

[66] Vaxman I., Gertz M.A. (2022). How I approach smoldering multiple myeloma. Blood.

[67] Soliman A.M., Das S., Teoh S.L. (2021). Next-Generation Biomarkers in Multiple Myeloma: Understanding the Molecular Basis for Potential Use in Diagnosis and Prognosis. Int J Mol Sci..

[68] Manier S., Park J., Capelletti M., Bustoros M., Freeman S.S., Ha G. (2018). Whole-exome sequencing of cell-free DNA and circulating tumor cells in multiple myeloma. Nat. Commun..

[69] Alipour E., Pooyan A., Shomal Zadeh F., Darbandi A.D., Bonaffini P.A., Chalian M. (2023). Current Status and Future of Artificial Intelligence in MM Imaging: A Systematic Review. Diagnostics.

[70] Allegra A., Tonacci A., Sciaccotta R., Genovese S., Musolino C., Pioggia G., Gangemi S. (2022). Machine Learning and Deep Learning Applications in Multiple Myeloma Diagnosis, Prognosis, and Treatment Selection. Cancers.

[71] Neri P., Lee H., Bahlis N.J. (2024). Artificial Intelligence Individualized Risk Classifier in Multiple Myeloma. J. Clin. Oncol..

[72] Fan G., Cui R., Zhang R., Zhang S., Guo R., Zhai Y., Yue Y., Wang Q. (2022). Routine blood biomarkers for the detection of multiple myeloma using machine learning. Int. J. Lab. Hematol..

[73] Kotter E., Ranschaert E. (2021). Challenges and solutions for introducing artificial intelligence (AI) in daily clinical workflow. Eur. Radiol..

[74] Kyle R.A., Rajkumar S.V. (2010). Monoclonal gammopathy of undetermined significance and smoldering multiple myeloma. Curr. Hematol. Malig. Rep..

[75] Landgren O., Rajkumar S.V. (2016). New Developments in Diagnosis, Prognosis, and Assessment of Response in Multiple Myeloma. Clin. Cancer Res..

[76] van Nieuwenhuijzen N., Spaan I., Raymakers R., Peperzak V. (2018). From MGUS to Multiple Myeloma, a Paradigm for Clonal Evolution of Premalignant Cells. Cancer Res..

[77] Bolli N., Avet-Loiseau H., Wedge D.C., Van Loo P., Alexandrov L.B., Martincorena I., Dawson K.J., Iorio F., Nik-Zainal S., Bignell G.R. (2014). Heterogeneity of genomic evolution and mutational profiles in multiple myeloma. Nat. Commun..

[78] Mittelman M., Israel A., Oster H.S., Leshchinsky M., Ben-Shlomo Y., Kepten E., Dolberg O.J., Balicer R., Shaham G. (2025). Can we identify individuals at risk to develop multiple myeloma? A machine learning-based predictive model. Br. J. Haematol..

[79] Wang M., Yu Y.-C., Liu L., Schoen M.W., Kumar A., Vargo K., Colditz G., Thomas T., Chang S.-H. (2023). Natural Language Processing–Assisted Classification Models to Confirm Monoclonal Gammopathy of Undetermined Significance and Progression in Veterans’ Electronic Health Records. JCO Clin. Cancer Inform..

[80] Hashemi Z.S., Khalili S., Malaei F., Mard-Soltani M., Jafarisani M., Lotfi J., Deemeh M.R., Zakeri A., Rasaee M.J. (2019). Serum DKK1 is correlated with γ peak of serum electrophoresis in multiple myeloma: A multicenter biomarker study. Biomark. Med..

[81] Gerov V., Gerova D., Micheva I., Nikolova M., Mihaylova G., Galunska B. (2023). Dynamics of Bone Disease Biomarkers Dickkopf-1 and Sclerostin in Patients with Multiple Myeloma. J. Clin. Med..

